# Innovative Product Design Based on Comprehensive Customer Requirements of Different Cognitive Levels

**DOI:** 10.1155/2014/627093

**Published:** 2014-06-09

**Authors:** Xiaolong Li, Wu Zhao, Yake Zheng, Rui Wang, Chen Wang

**Affiliations:** ^1^School of Manufacturing Science and Engineering, Sichuan University, First Ring Road, No. 24, Chengdu, Sichuan 610065, China; ^2^School of Electrical Engineering and Computer Science, University of Ottawa, 75 Laurier Avenue East, Ottawa ON, Canada K1N 6N5

## Abstract

To improve customer satisfaction in innovative product design, a topology structure of customer requirements is established and an innovative product approach is proposed. The topology structure provides designers with reasonable guidance to capture the customer requirements comprehensively. With the aid of analytic hierarchy process (AHP), the importance of the customer requirements is evaluated. Quality function deployment (QFD) is used to translate customer requirements into product and process design demands and pick out the technical requirements which need urgent improvement. In this way, the product is developed in a more targeted way to satisfy the customers. the theory of innovative problems solving (TRIZ) is used to help designers to produce innovative solutions. Finally, a case study of automobile steering system is used to illustrate the application of the proposed approach.

## 1. Introduction

In today's rapidly evolving global and fiercely competitive economic environment, many companies are conscious of the significance of product innovation. A key factor in the model of creativity for product innovation is design methodology [1]. With this in mind, many practitioners and academic researchers suggested many principles and approaches to enhance the quality and efficiency of innovation, such as lead users [2], fuzzy front end (FFE) [3], quality function deployment (QFD) [4], axiomatic design theory (ADT) [5], function structure method [6], and TRIZ [7].

QFD is a systematic customer-drive product design method for translating the voice of the customer through the various stages of product planning, engineering, and manufacturing [8]. The first phase of QFD is a matrix called House of Quality (HoQ), in which the customer requirements are identified and translated into technical requirements. HoQ has fundamental and strategic importance in the QFD system, so it is often used as the framework to integrate other approaches, such as ADT [9, 10] and TRIZ [11, 12].

Many researchers have done a great deal of work from different perspectives to improve QFD since it was conceived in Japan in the late 1960s. Generally, the product development process consists of four steps in QFD.

**Step 1**

Acquire and systemize customer requirements.

**Step 2**

Determine the weight of customer requirements.

**Step 3**

Translate the customer requirements into technical requirements and determine which should be improved.

**Step 4**

Develop the product with consideration of the selected technical requirements.

A lot of research has been conducted according to the above steps.

Various methods have been applied to acquire customer requirements, which are usually divided into two main categories: technology driven and market driven. Technology driven predicts the evolution of the customer requirements based on the analysis of function requirement patterns of previous products and its most popularly used theory is TRIZ evolution theory. Market driven includes many powerful tools such as 5W1H analysis, tracing method, focus group, individual interviews, affinity diagram, and cluster analysis [13, 14]. These methods are mature and widely used. However, the methods mentioned above are often operated subjectively and the quality of the acquisitions depends on the proficiency of the interviewers.

Determining the weight of customer requirements is a critical step in QFD, so considerable effects should be committed to rank the customer requirements correctly. There are many methods available to determine the weight of customer requirements. The simplest method is the point scoring scale. However, the score is often obtained from survey in which many customers tend to rate every requirement to an extreme score, which may make this method ineffective. Akao, the father of QFD, has suggested the application of the AHP in prioritizing the customer requirements in QFD. AHP has been considered much better than the traditional approach, such as using a scale of 1–5, because it can provide ratio scale priorities and judgment's consistency check. Due to its simplicity and great flexibility, AHP has been found to be one of the most popular tools applied in QFD in the last decade [15–17]. Taking the interdependence among customer requirements into account, analytic network process (ANP), as an AHP's variant, has increasingly been used recently [18]. Some methods, such as fuzzy set and rough set theory, have been used to handle the vagueness and uncertainty in the product development process [16, 19]. In order to utilize their respective advantages, some combinative approaches have been developed, such as fuzzy AHP and fuzzy ANP.

Translating the customer requirements into technical requirements and determining which technical requirements should be improved may be a complicated decision-making process, as it involves not only the customers but also the competitors. In this respect, the sales point concept has received the most attention to take competitors into consideration [20]. As a concept in information theory, entropy method can measure the expected information content of a certain message and has been employed to integrate competitive analysis into product design and development in QFD [20–22]. To understand the voice of customer deeply in an economic way, Kano's model has also been incorporated into the decision-making process by classifying the customer requirements into three categories [16, 23].

After customer requirements are translated into technical requirements, a company develops the product purposely based on the ratings of technical requirements. The primary mission of HoQ is facilitating the identification of improvement opportunities, and it is almost accomplished at this step. Then specific solutions can be obtained by the various innovative methodologies mentioned in the beginning. QFD can not only rate the technical requirements to get the targeted improvement, but also reveal the relationship between the technical requirements in the roof of HoQ. And TRIZ is an appealing tool to deal with the information in HoQ, which has been applied in numerous industries among researchers and practitioners.

Previous research has facilitated the development of the user-centered innovative product design from different perspectives. However, there are still some deficiencies. This paper mainly discusses the following aspects. (1) Little attention has been devoted to capture the customer requirements in a systematic way. So it is hard to acquire the customer requirements comprehensively. Besides, the organization and expression of requirements are disordered, which may bring difficulty in sharing information. (2) When applying QFD, the requirements in consideration are mostly related to function, while the requirements beyond function are ignored. (3) The studies of rating customer requirements focus on the operation method itself, rather than thinking about this problem from a practical perspective.

Aiming at the problems mentioned above, this paper studies the following aspects. (1) Customer requirements, including functional requirements and other requirements beyond function, are analyzed in a comprehensive way. Based on the research of the relationship between different levels of demand, a topology structure of customer requirements is established. (2) A method of using and extending the topology structure is presented and on this basis the importance of the customer requirements is evaluated with the assistance of AHP. (3) Considering the factors of existing products and markets, the customer requirements are translated into technical requirements by QFD. And the technical requirements which should be improved are picked out according to the results of the calculation. (4) Based on the analysis of the requirements, TRIZ is employed to generate the innovative solution.

## 2. Method for Capturing Requirements

### 2.1. Comprehensive Customer Requirements

With the rapid development of productive forces, people's living standard has got a great improvement. So people are no longer satisfied with the products which can just fulfil functional tasks. In other words, consumers' purchase decisions are influenced by other factors, such as appearance, aesthetics, affection, usability, and comfortableness. However, due to the restrictions of designers' knowledge and thought patterns, traditional design methodologies often ignore those factors, which may be the fountainhead of some ugly or troublesome products.

Products should exist not only to perform tasks, but also to satisfy other requirements, which are called soft requirements [24]. While it is acceptable that functionalities are crucial for products' success, the soft requirements have been paid increasing attention in today's complex and changeable marketplace.

The early research about soft requirements has concentrated on the visual factors [25], which is significant but insufficient. Some research has been extensively carried out to increase the user friendliness of the product and some progress has been made in the field of ergonomics. In order to prompt customers to buy the products, some methods have been developed to “design for wow,” which mean conferring a sense of excitement to the product [26]. Kansei Engineering has provided a systematic approach to measure customers' feelings and impressions and then translate them into product parameters, which is a useful methodology in handling consumers' emotional requirements [27].

In order to consider all the factors scientifically, the customer requirements and product features have been divided into several levels of hierarchy from different perspectives. A. H. Maslow's hierarchical theory has penetrated into the area of product design and the customers' needs have been classified into three categories of functionality, usability, and pleasure [28]. The development process of product has been divided into three levels: holistic attributes, functional design, and styling design [29]. Considering the interaction between users and products, a taxonomy modeling strategy has been defined, including the three levels of aesthetic, meaning, and emotional [30].

Based on the study of the cognitive psychology, a classification is proposed to understand consumer requirements comprehensively, which is called emotional design classification (EDC) in this paper. The relevant requirements are also obtained according to the different cognitive levels. The aim of this research is to gain a deeper insight into customer requirements and integrate these requirements into the innovative product design process. In subsequent sections, the details of the EDC as well as corresponding requirements are discussed and a topology structure is established based on EDC.

### 2.2. Emotional Design Classification and Requirements

Emotion is a kind of human physiological reaction caused by the outside world, which is determined by people's needs and expectations. When the needs and expectations are met, a pleasant emotion will arise. On the contrary, an afflictive emotion will emerge. In EDC the human cognition process is divided into visceral, behavioral, and reflective levels [31]. Every cognitive level works in a different way. Therefore, each cognitive level has its corresponding requirements in the process of product design.

The visceral cognition is the feeling one gets when first encountering a product, which mainly comes from the sensory organs. The visceral cognition impacts emotion directly rather than through consciousness, so it reacts quickly and does not differ due to acquired factors such as education and culture. The behavioral cognition is the feeling one gets when using a product, which mainly comes from the motor organs. The behavioral cognition has few dependencies on consciousness. For example, most people can ride a bike while thinking about other things. The reflective cognition is the highest level of cognitive process, which is the brain's introspection of the visceral and behavioral cognition. The reflective cognition is concerned with the information and other factors such as the meaning of products, which is quite different due to cultural diversity.

These three levels of cognition are interdependent. The visceral cognition can quickly capture some information of products and pass this information to the motor organs directly without the processing of the brain. For instance, in emergency, humans have an ability to avoid danger instinctively rather than taking action after thinking it over. In turn, the behavioral cognition will also impact on visceral cognition. While there is no connection channel between the reflective cognition and the outside world, reflective cognition receives information from the visceral and behavioral cognition and adversely affects the visceral and behavioral cognition after processing this information.

**Figure 1:**
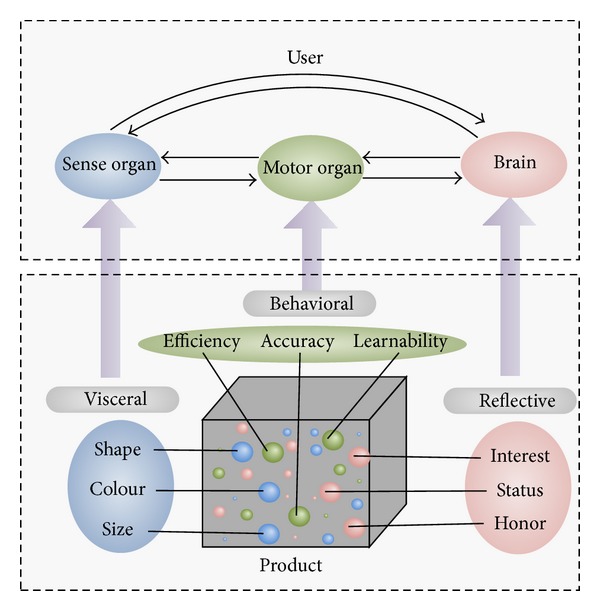
Emotional design classification and requirements.

The requirements are classified according to different cognitive levels, as shown in Figure 1. The visceral requirements refer primarily to the appearance design of products, which also include auditory, somatosensory, and some other factors. Since the response speed of visceral cognition is very rapid, it can largely influence users' buying decision. The behavioral requirements are an overarching concern in traditional design. However, the behavioral requirements are endowed with some higher demands in the new design process. In the first place, the product should perform its function effectively. On the other hand, the customer's feeling also needs to be considered. Learnability and usability are important criteria to measure the quality of products in the behavior level [32]. The reflective requirements are related to the meanings of the products, which are affected by the environment, culture, identity, and so on. In order to improve customer loyalty, reflective requirements should be satisfied based on the analysis of the customers and their social groups. The accurate product positioning is the key to solve this complex and volatile problem.

In order to make products get consumers' recognition, it is necessary to try to meet the consumer requirements in all levels of the design process. In actual situation, however, due to the boundaries between disciplines, it is difficult to take all aspects into account. Aiming at this problem, a topology structure of customer requirements is established based on EDC.

### 2.3. Topology Structure of Customer Requirements Based on EDC

The establishment process of topology structure of customer requirements is as follows.

**Step 1**

Collect terms associated with the customer requirements from some design cases in different industries. By analyzing these collected terms, it is found that the expression and subdivision degree are inconsistent for the same requirement, so standardization of these terms is essential.

**Step 2**

Establish the framework of topology structure. Classify the collected terms according to the correlation and the degree of abstraction and choose some typical terms to build the main framework of topology structure.

**Step 3**

Complete the topology structure. Fill the remaining terms in the framework after unifying their expression.

**Step 4**

Supply and modify the topology structure in the process of repeated use.

**Figure 2:**
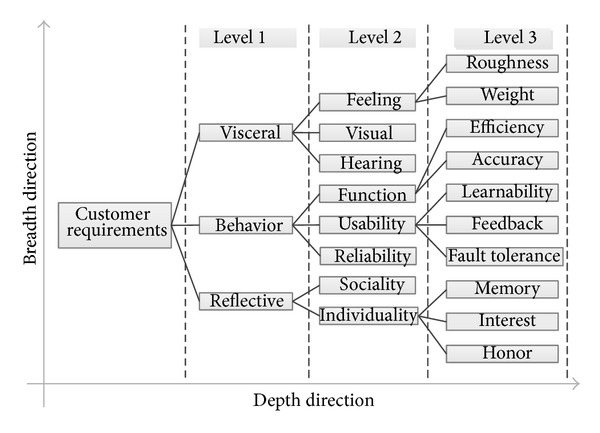
Topology structure of customer requirements.

Finally the topology structure of customer requirements is established as shown in Figure 2. The existing topology contains three levels in subdivision degree. The first level is based on EDC, namely, visceral, behavioral, and reflective requirements. The terms in the second level can describe products in different fields, which are the subdivision of the terms in the first level. The third level is the further subdivision of the second level. Requirements in the third level are different from product to product. In actual application process, the topology structure is extended from both depth and breadth direction. The extending strategy is detailed in the following section. Topology structure of customer requirements develops the traditional design method into a new design method, in which both functional requirements and soft requirements are considered.

## 3. Innovative Product Design Based on Requirements

**Figure 3:**
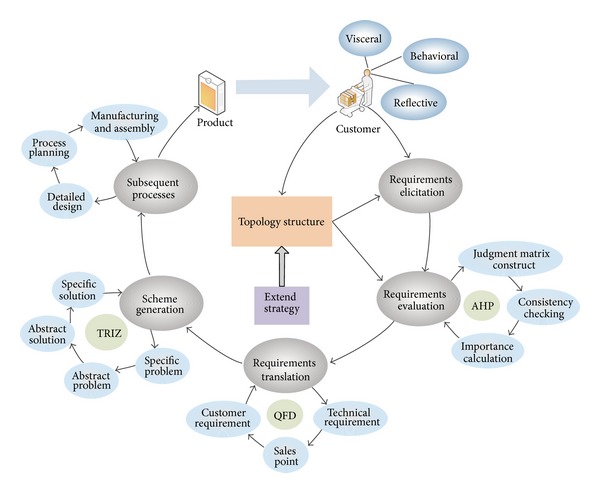
Demand-driven product innovation design process.

In order to have a comprehensive analysis of customer requirements and infuse these requirements into design process, this paper presents a demand-driven innovative product design process, which is composed of four stages, namely, requirements elicitation, requirements evaluation, requirements translation, and scheme generation. In the first stage, the customer requirements are elicited with the aid of the topology structure of customer requirements and its corresponding extending strategy. In the second phase, based on the sorting thought in EDC, the relative importance of the customer requirements is evaluated by AHP. In the third stage, taking into account the existing products and market competition factors, the customer requirements are translated into the technical requirements using HoQ, which is the principal tool of QFD. After that, the technical requirements are ranked according to the importance. In the fourth stage, TRIZ is used to solve the problems and generate innovative solutions according to the importance and relationship of the technical requirements. The demand-driven innovative product design process is shown in Figure 3. As can be seen from the figure, the whole process forms a complete circle, which starts from the customers and ends in the products. All of the work revolves around the requirements so that the final design product can satisfy the customers.

### 3.1. Requirements Elicitation

**Figure 4:**
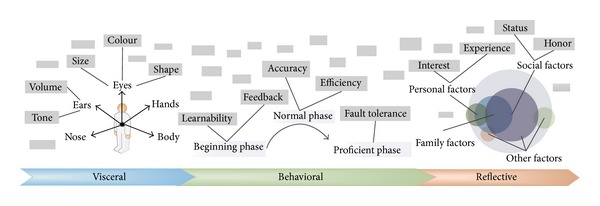
Extended strategy of topology structure of customer requirements.

In the process of eliciting customer requirements, designers choose related terms in the topology structure of customer requirements and embody the terms according to different products. Designers should use the topology structure as a guide flexibly and extend the topology structure according to the actual situation, rather than being restricted by the existing terms. The extending strategy of the topology structure includes sensory organs extending, using process extending, and group extending, as shown in Figure 4.

Sensory organs extending is the strategy that extends the requirements from human being's sensory organs, which is mainly used to analyze the visceral requirements. Visceral cognitions come from sensory organs, so the visceral requirements can be obtained more comprehensively in this way. Specific steps are as follows. First, list the sensory organs which can receive signals from products. Then, mine other information about products according to the various sensory organs, respectively. Consider the example of eyes, which are one of the sensory organs and can receive signals, like color, size, and shape. These signals are factors that may generate requirements, which need careful consideration in the design process. In addition, besides some common sensory organs such as eyes, ears, and hands, some other internal sensory organs and comprehensive factors should also be taken into account, such as weightlessness, spinning sensation, and sense of balance. These factors also come from visceral cognition, but it is difficult to determine which sensory organs generate these factors. In the design process, it is a challenging and meaningful work.

Using process extending is the strategy that extends the requirements from different phases in the whole using period, which is mainly used to analyze the behavioral requirements. The whole using period is divided into beginning phase, normal phase, and proficient phase. When extending the behavioral requirements, customers belong to different phases rather than the single phase which should be observed and analyzed. In the beginning phase, some issues about how to make the product easy to use are considered. Because the customers are unfamiliar to the new products, it is necessary to provide some hints to guide the users. In the normal phase, the functions that products should perform are the primary focus. Some correlative factors, such as durability and failure rate, should also be considered. In the proficient phase, as the users have become more proficient in operating the products, the dependence on consciousness gradually reduced. So some efforts should be made to ensure the safety and reliability of the unconscious operation. In addition, more attentions need to be paid to make products more enjoyable to use.

Group extending is the strategy that extends the requirements from the customers' groups, which is mainly used to analyze the reflective requirements. Humans are not only products of nature, but also creatures of society. The reflective requirements differ from environments. Some groups may be taken into consideration, such as individuals, families, and society. For example, from an individual viewpoint, hobbies and experiences may affect the reflective requirements. However, a product becomes a symbol of identity and honor in social environment.

### 3.2. Requirements Evaluation

After requirements elicitation, the customer requirements are evaluated so that more important requirements can be selected.

When evaluating the first-level requirements, which consist of visceral, behavioral, and reflective requirements, products are subdivided based on how the requirements affect the sales. When one aspect of requirements is met, the growth degree of sales differs depending upon the types of products. For instance, in terms of some lower price products, if the visceral requirements are improved, the sales may boost greatly. In this case, the customers spend less time on the purchasing behavior, so the visceral satisfaction is sufficient to promote the deal.

When evaluating the visceral requirements, products are subdivided based on the amount of information received by the sensory organs. In many cases, amount of information obtained by eyes is the largest so the visual requirement is often the most important. When evaluating the behavioral requirements, products are subdivided based on the proportion of the users. For example, the learnability is more important than efficiency for a product in public place, of which the major user groups are in the beginning phase, so it may take longer time to learn how to use it than actually use it. When evaluating the reflective requirements, products are subdivided based on the environment in which the products are used. For example, when designing a private product, a wonderful memory is more important than the sense of honor.

In this paper, the aforementioned sorting thought is implemented by AHP because of its less cumbersome mathematical calculations and comprehensibility. AHP is a multicriteria decision method, which is originated by Saaty in the nineteen seventies [33]. In the application of AHP, the indicators in the problem are arranged in a hierarchical structure model, and the relative importance of each factor to the upper level factor is obtained. The steps of applying AHP are discussed as follows.

**Step 1**

Establish the judgment matrix. The indicators, which belong to the same indicator of the higher level, are compared with each other. The results are expressed by the 9-point scale. The established matrix is expressed as

(1) $$\begin{array}{c} A=\left[\begin{array}{ccc} a_{11} & \cdots & a_{1n}\\ \vdots & \ddots & \vdots \\ a_{n1} & \cdots & a_{nn} \end{array}\right]. \end{array}$$

Here, *a*_*ij*_ stands for the comparison between the indicator *i* and *j*, of which the meanings are shown in Table 1.

**Step 2**

Check the consistency of the matrix to guarantee that the decision makers' judgments are consistent. The consistency ratio *C*_*R*_ is the indicator to measure the consistency of the judgment matrix. *C*_*R*_ is calculated by the following formula:

(2) $$\begin{array}{c} C_{R}=\frac{\lambda_{\max}-n}{\left(n-1\right)R_{I}}. \end{array}$$

Here, *λ*_max⁡_ is the maximum eigenvalue of the matrix and *n* is the order of the matrix. *R*_*I*_ is random index, which depends on *n*, as show in Table 2. When *C*_*R*_ < 0.1, the judgment matrix is accepted. Otherwise, the judgment matrix should be revised.

**Step 3**

Rank indicators in single hierarchy. Eigenvalue method is used to get the weight vectors by the following formula:

(3) $$\begin{array}{c} \left(A-\lambda_{I}\right)W=0. \end{array}$$

Here, *λ* is one of the eigenvalues of the matrix and *W* is the eigenvectors corresponding to the eigenvalues *λ*. *W* is also the weights vector.

**Step 4**

Rank indicators in the whole system. After the weights of the indicators in each hierarchy are obtained, multiply all weights together from bottom to top hierarchy, and the result is the total weight.

### 3.3. Requirements Translation

**Figure 5:**
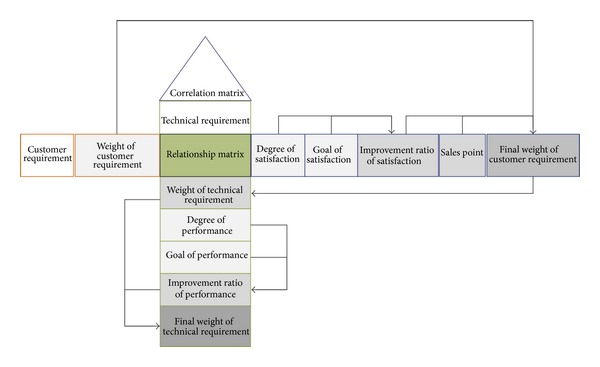
The sketch of the House of Quality.

After being analyzed, customer requirements are translated into technical requirements by HoQ, so that the technical requirements which need to be improved are picked out. The HoQ is shown in Figure 5.

Customer requirements, *C* = (*c*_1_, *c*_2_, …, *c*_*n*_), and their corresponding weights, *W*_*C*_ = (*w*_*c*1_, *w*_*c*2_, …, *w*_*cn*_), are in the left wall of HoQ. Technical requirements, *T* = (*t*_1_, *t*_2_, …, *t*_*m*_), are in the ceiling. The relationship matrix of the customer requirements and technical requirements is in the middle of the house, which can be expressed as

(4) $$\begin{array}{c} R=\left[\begin{array}{ccc} r_{11} & \cdots & r_{1m}\\ \vdots & \ddots & \vdots \\ r_{n1} & \cdots & r_{nm} \end{array}\right]. \end{array}$$

Here, *r*_*ij*_(0 < *i* ≤ *n*, 0 < *j* ≤ *m*) stands for the relevancy between *c*_*i*_ and *t*_*j*_, of which the value range is 0–9. There are some factors about the customer satisfaction on the right side of the relationship matrix. From left to right, they are degree of satisfaction, *D*_*s*_ = (*d*_*s*1_, *d*_*s*2_, …, *d*_*sn*_), goal of satisfaction, *G*_*s*_ = (*g*_*s*1_, *g*_*s*2_, …, *g*_*sn*_), and improvement ratio of satisfaction, *I*_*s*_ = (*i*_*s*1_, *i*_*s*2_, …, *i*_*sn*_). *I*_*s*_ can be calculated by the following formula:

(5) $$\begin{array}{c} i_{si}=\frac{g_{si}}{d_{si}}. \end{array}$$

Sales point, *S* = (*s*_1_, *s*_2_, …, *s*_*n*_), indicates the unique selling position to separate one's own products from their competitors. Higher sales point can be found among the areas that competitors perform poorly. The most commonly used values are 1, 1.25, and 1.5, corresponding poor sales point, moderate sales point, and strong sales point, respectively [20]. The final weight of customer requirements, FW_*c*_ = (fw_*c*1_, fw_*c*2_, …, fw_*cm*_), can be calculated by the following formula:

(6) $$\begin{array}{c} \text{f}{\text{w}}_{ci}=w_{ci}\cdot i_{si}\cdot s_{i}. \end{array}$$

The floor of the HoQ is some factors related to the technical requirements. From top to bottom, they are weight of technical requirements, *W*_*t*_ = (*w*_*t*1_, *w*_*t*2_, …, *w*_*tm*_), which can be calculated by the following formula:

(7) $$\begin{array}{c} w_{tj}=\overset{n}{\underset{i=1}{\sum }}r_{ij}\times \text{f}{\text{w}}_{ci}. \end{array}$$

Degree of performance *D*_*p*_ = (*d*_*p*1_, *d*_*p*2_, …, *d*_*pm*_), goal of performance *G*_*p*_ = (*g*_*p*1_, *g*_*p*2_, …, *g*_*pm*_), and improvement ratio of performance *I*_*p*_ = (*i*_*p*1_, *i*_*p*2_, …, *i*_*pm*_), which can be calculated by the following formula:

(8) $$\begin{array}{c} i_{pj}=\frac{g_{pj}}{d_{pj}}. \end{array}$$

Final weight of technical requirements FW_*t*_ = (fw_*t*1_, fw_*t*2_,…, fw_*tm*_), which can be calculated by the following formula:

(9) $$\begin{array}{c} \text{f}{\text{w}}_{tj}=w_{tj}\times i_{pj}. \end{array}$$

The roof of the HoQ is the correlation matrix of the technical requirements. Positive and negative sign are often used to stand for the positive and negative correlation, respectively.

### 3.4. Scheme Generation

After analyzing the consumer and technical requirements, TRIZ theory is used to solve the problem. TRIZ theory suggests that creative problem contains at least one contradiction. Resolving contradiction is the driving force of development, which is also an important step to solve problem [7]. The main tools of TRIZ include the contradiction matrix, substance-field analysis, the ideal final result, and effect knowledge base.

For the proposed method, there are two ways to improve the product. One way is to emphasize improving the technical requirements with high fw_*t*_ in HoQ. According to the above analysis, the technical requirements with high fw_*t*_ have closer relationship with consumer requirements and can be improved effectually with the consideration of the current markets factors and technical ability. The other way is to analyze the relevance of the technical requirements in the correlation matrix and then identify and resolve the contradiction so that several technical requirements can be optimized simultaneously.

## 4. Case Study

Automotive steering system is one of the components the drivers contact most frequently, which plays an important role in driving safety and fun. And it is also a common product in daily life, so it is chosen to be the case of the proposed method. The specific procedure is as follows.

### 4.1. Requirements Elicitation

**Figure 6:**
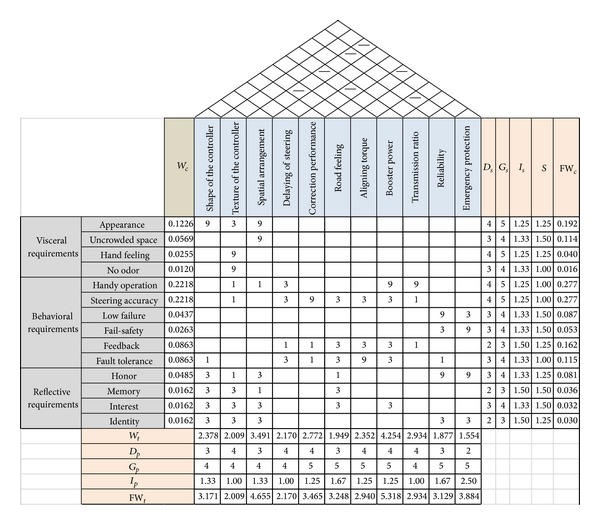
The HoQ for steering system.

First, the visceral requirements are considered. Appearance and uncrowded space are the requirements on the visual aspects and hand feeling is the requirement on feeling, which can be found in the topology structure. Based on visual and feeling, the smelling requirement can be extended by the sensory organs extending. And embodying it, the requirement of no odor is obtained. Then the behavioral requirements are analyzed. In the beginning phase, feedback of operation is selected. In the normal phase, accuracy and efficiency are the subitems of function, which can be, respectively, specialized as steering accuracy and handy operation. In view of the reliability, both low failure rate and the fail-safety should be considered. In the proficient phase, the fault tolerance is obtained. Finally, considering the service environment of the steering system, reflective requirements are obtained by group extending. The reflective requirements consist of personal interest, memory, identity, and honor. All the requirements are filled in the left wall of the HoQ, as shown in Figure 6.

### 4.2. Requirements Evaluation

According to the method mentioned above, the judgment matrices are obtained, which are shown as follows:

(10) A1=[113331513151],A21=[1357131351513131715131],A22=[1157331157331515131313171713113131313331113133311],A23=[1333131111311113111].

The corresponding criteria of *A*_1_ are visceral requirements, behavioral requirements, and reflective requirements in the first level of customer requirements. There are three matrices in the second level of customer requirements. The corresponding criteria of *A*_21_ are appearance, uncrowded space, hand feeling, and no odor, which belong to visceral requirements. The corresponding criteria of *A*_22_ are handy operation, steering accuracy, low failure, fail-safety, feedback, and fault tolerance, which belong to behavioral requirements. The corresponding criteria of *A*_23_ are honor, memory, interest, and identity, which belong to reflective requirements.

To verify the rationality of the judgment matrices, the results of the consistency check are in Table 3.

It can be seen from the table that each index of *C*_*R*_ values is less than 0.1, so the indicators are consistent. The indicator weights of each layer are multiplied to get the total weights, and then the results are normalized and filled in the left wall of the HoQ.

### 4.3. Requirements Translation

Customer requirements, technical requirements, and some other factors are filled in the HoQ, as shown in Figure 6.

As can be seen from the calculations, road feeling, booster power, reliability, spatial arrangement, and some other technical requirements have high fw_*t*_. In addition, several contradictions can be found in the correlation matrix, such as reliability and booster power, emergency protection, and road feeling, as shown in the roof of the HoQ.

### 4.4. Scheme Generation

The existing steering systems mainly include mechanical steering system, hydraulic power steering system, electric power steering system, and the steering-by-wire system. Among them, the steering-by-wire system is the main developing trend of future automotive steering systems. Combining the analysis of HoQ, the steering-by-wire system has big advantages in the booster power and spatial arrangement. However, there are still some defects in other aspects, such as road feeling, reliability, and emergency protection. To solve this problem, the substance-field analysis of TRIZ is employed to improve the steering-by-wire system.

**Figure 7:**
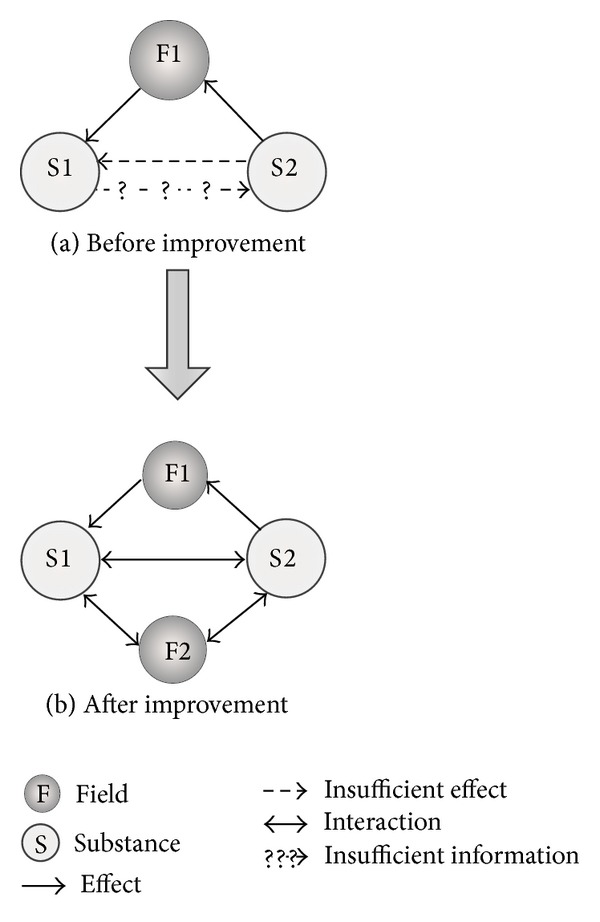
Substance-field model of steering system.

The substance-field model of the steering-by-wire system is shown in Figure 7(a). In the figure, S1 is steering wheel, S2 is the controller, and F1 is the steering system. Due to the elimination of the mechanical connection, the execution of steering movement only depends on wire device, which is defective. First, when the device fails, the steering operation cannot be performed, which is expressed as insufficient effect in the substance-field model. Second, the drivers cannot receive road feeling from the wheel, which is expressed as insufficient information in the substance-field model. Therefore, the solution of introducing a new field to evolve the substance-field model to parallel type in 76 standard solutions is selected to solve the problem. Considering the spatial arrangement, the new introduced field should be flexible. Original system introduces a new field of F2, which is a set of hydraulic devices. In the improved substance-field model of steering system, the interaction between S1 and S2 is sufficient under the action of F2, as shown in Figure 7(b).

**Figure 8:**
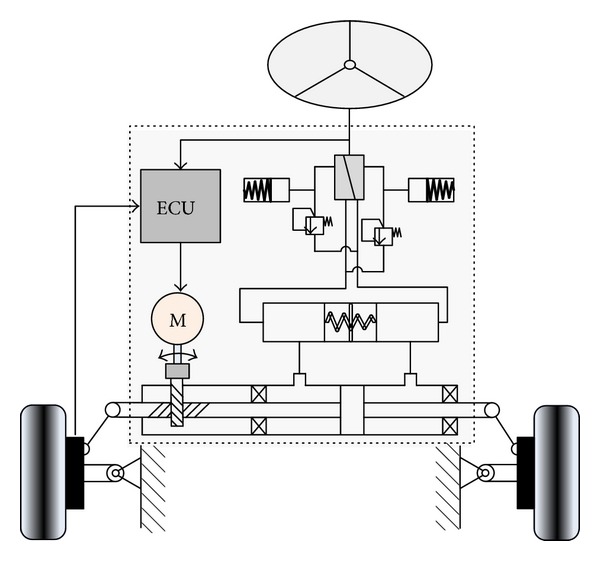
The improved steering-by-wire system.

The improved steering system has the following characteristics. (1) In the aspect of spatial arrangement, it inherits the merit of the ordinary steering-by-wire system due to the flexibility of the hydraulic device. (2) Hydraulic device provides the road feeling, which is a real feeling rather than a feeling simulated by the motor. (3) Aligning torque provided by the hydraulic device is more reliable than that provided by the electronic component. (4) When the steering-by-wire system fails, the hydraulic device can execute the task of steering temporarily, which provides a reliable emergency protection. The improved steering-by-wire system is shown in Figure 8.

## 5. Conclusions

In this paper, according to the human cognitive processes, the customer requirements are classified into visceral, behavioral, and reflective requirements. Based on the classification, a topology structure of customer requirements is established and corresponding extending strategy is proposed. The customer requirements, which include not only functional requirements but also soft requirements, can be obtained comprehensively with the help of the topology structure. A demand-driven product innovation design process is proposed based on the topology structure of customer requirements. AHP is used to evaluate the importance of the obtained requirements. QFD is a framework for the whole design process, which translates the customer requirements into technical requirements. Designers can improve the product in a more targeted way by analyzing the results of HoQ. TRIZ theory is used to solve the problem in the conceptual design process, which leads designers to generate innovative solutions. Finally, the proposed method is used to design an automobile steering system. The design scheme meets the customer requirements from different perspectives, which is achievable in the current technical condition. Therefore, the design method proposed in this paper is valid.

However, since the main research content of this paper focuses on the conceptual design phase, the research on detailed design phase is insufficient. In the following work, some theories and methods regarding design collaboration should be combined with the existing research so that the requirements in different levels can be met during the detailed design phase in a teamwork environment. In addition, future work can also be done to introduce some other classification methods, such as Kano model, into this method to develop the topology structure of customer requirements into multidimensional structure.

## Figures and Tables

**Table 1:** Value of judgment matrix.

Intensity of importance	Explanation
1	Equally important
3	Moderately more important
5	Strongly more important
7	Very strongly more important
9	Extremely more important
2, 4, 6, and 8	Intermediate values
Reciprocals of the above numbers	Inverse comparisons

**Table 2:** Random index.

*n*	1	2	3	4	5	*⋯*
*R*_*I*_	0	0	0.52	0.89	1.12	*⋯*

**Table 3:** Results of the consistency check.

	*A*	*A*_21_	*A*_22_	*A*_23_
*λ*_max⁡_	3.0385	4.1170	6.1494	4.0000
*C*_*I*_	0.01925	0.039	0.02988	0
*R*_*I*_	0.52	0.89	1.26	0.89
*C*_*R*_	0.037	0.044	0.024	0
